# Toppling Pencils—Macroscopic Randomness from Microscopic Fluctuations

**DOI:** 10.3390/e22091046

**Published:** 2020-09-18

**Authors:** Thomas Dittrich, Santiago Peña Martínez

**Affiliations:** Departamento de Física, Universidad Nacional de Colombia, Bogotá 111321, Colombia; spenam@unal.edu.co

**Keywords:** randomness, fluctuations, double well, bistability, finite heat bath, relaxation, irreversibility, chaos, spin-boson model

## Abstract

We construct a microscopic model to study discrete randomness in bistable systems coupled to an environment comprising many degrees of freedom. A quartic double well is bilinearly coupled to a finite number *N* of harmonic oscillators. Solving the time-reversal invariant Hamiltonian equations of motion numerically, we show that for N=1, the system exhibits a transition with increasing coupling strength from integrable to chaotic motion, following the Kolmogorov-Arnol’d-Moser (KAM) scenario. Raising *N* to values of the order of 10 and higher, the dynamics crosses over to a quasi-relaxation, approaching either one of the stable equilibria at the two minima of the potential. We corroborate the irreversibility of this relaxation on other characteristic timescales of the system by recording the time dependences of autocorrelation, partial entropy, and the frequency of jumps between the wells as functions of *N* and other parameters. Preparing the central system in the unstable equilibrium at the top of the barrier and the bath in a random initial state drawn from a Gaussian distribution, symmetric under spatial reflection, we demonstrate that the decision whether to relax into the left or the right well is determined reproducibly by residual asymmetries in the initial positions and momenta of the bath oscillators. This result reconciles the randomness and spontaneous symmetry breaking of the asymptotic state with the conservation of entropy under canonical transformations and the manifest symmetry of potential and initial condition of the bistable system.

## 1. Introduction

For not few of its pioneers, the theory of deterministic chaos came with the hope for a deterministic description of an important part of physical phenomena, which till then had been relegated to the realm of randomness. It rapidly became clear, however, that in many areas, random processes would remain an indispensable element of the theoretical analysis. Deterministic chaos had to be brought together with noise again [1], criteria were developed to distinguish chaos from mere chance [2], and with the subject of quantum chaos, the question was addressed how deterministic chaos could be modified to reconcile it with a theory considered as fundamentally probabilistic.

At the same time, important paradigms of chance in macroscopic phenomena remain that defy an understanding in terms of deterministic chaos. A fascinating particular instance is randomizing devices in games of luck, such as tossed coins [3], dreidels, dice, or roulette wheels [4]. Their underlying dynamics is not chaotic, it is rather the discretization of the final condition into two, four, six, or 37 bins that results in a sensitive dependence on the initial condition and thus reduces a continuous angle coordinate to a practically unpredictable integer. Notwithstanding, a description in terms of deterministic equations of motion is possible and allows for example to verify or falsify the presence of biasses in the outcomes.

Much more relevant from a physical point of view are processes that magnify microscopic dynamical disorder in many-body systems to randomness on macroscopic scales. A prototype of this phenomenon is Brownian motion, where the trajectory of a pollen grain amplifies thermal noise to direct observability. Traditionally, Brownian motion is represented as a stochastic process [5], using statistical descriptions such as Langevin or Fokker-Planck equations [6], without any more detailed examination of the underlying microscopic mechanisms.

**Figure 1:**
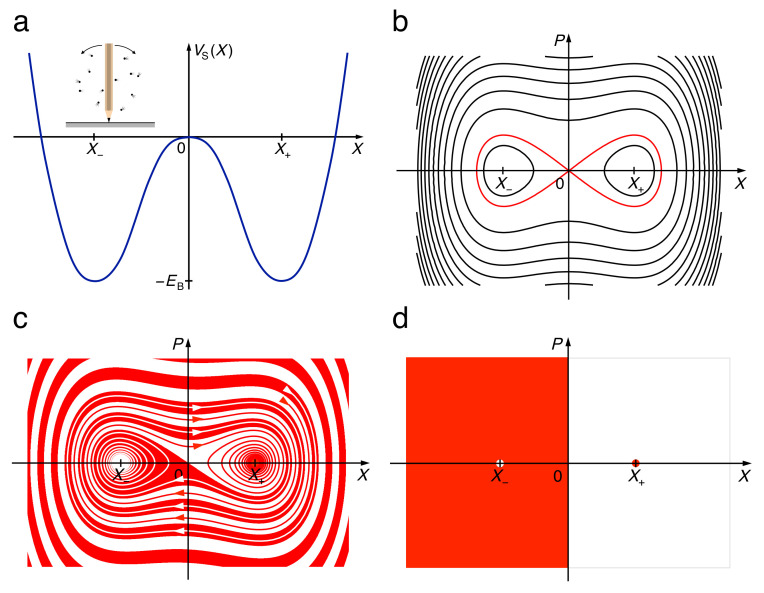
Hamiltonian motion (panels **a**,**b**), Equations (1) and (2), and dissipative dynamics (**c**,**d**), Equation (3), for a quartic double well. The potential (**a**), modelling a pencil balanced tip down on a flat surface (inset), shows two quadratic minima at $X_{\pm }$, related by parity X→−X, separated by a parabolic barrier with its top at $X_{0}=0$. Trajectories of the Hamiltonian dynamics (**b**) comprise approximately harmonic oscillations within each well for negative and strongly anharmonic oscillations for positive energies, separated by a separatrix (red) at E=0. Basins of attraction for weak friction (**c**) spiral around one another into either well. In the limit of strong friction (**d**), they coincide with the half spaces X<0 and X>0.

The present paper intends a synthesis of these two views of randomness, proposing a model that combines the discreteness of the output with the deterministic dynamics of a many-body system as random generator on the input side. The macroscopic central component, representing the tossed coin, is a bistable system, a symmetric double well modelled as a quartic oscillator. It can be seen as a physical representation of a classical bit, such as an inverted pendulum, or more graphically even, as a pencil balanced tip down on a flat surface (inset in Figure 1a).

The microscopic part adheres to the standard modelling of environments as heat baths, coupling the double well to a set of *N* harmonic oscillators. It is well known and has been argued in countless works in statistical mechanics, solid-state physics, and many other fields, that for N→∞ and under certain conditions on the frequency dependence of coupling and spectral density of the oscillators, the bath becomes an irreversible sink of information and energy, inducing relaxation to a stationary state and dissipation in the central system [7]. In this point, however, we adopt a more recent development in statistical mechanics, in that we keep the number of oscillators large, N≫1, *but finite* [8,9,10,11,12], so that the dynamics of the total system can be treated in the framework of the time-reversal invariant Hamiltonian mechanics of closed systems. It has been demonstrated for classical as well as for quantum systems [13,14], and is corroborated by the present work, that despite its time-reversal symmetry, this approach reproduces irreversible behaviour on all relevant timescales. Poincaré recurrences, which prevent true irreversibility in systems with a finite number of freedoms, occur only on timescales that diverge geometrically with *N* [15]. Our purpose, however, is not substantiating the approach to thermal equilibrium in these systems. Finite heat baths offer another advantage we exploit in the present context and which is excluded from the outset in an ensemble treatment: Fluctuations of the bath now become controllable and reproducible. This allows us to specify the initial conditions for each oscillator individually and in this way, to study how these fluctuations become manifest in the macroscopic randomness of the final state of the central system.

In particular, we would like to demonstrate that the outcome of this game of luck, whether the central system, initially prepared exactly in a “Buridan’s ass state”, the unstable equilibrium position on top of the barrier, falls into the left well (“tail”) or the right well (“head”), depends on asymmetries in the initial condition of the oscillators in the bath. Balance the pencil precisely tip down: If it still falls over, in which direction will it fall? It is determined by the environment, the particles of the surrounding gas impinging on the pencil. More generally, the bistable system amplifies and thus measures random fluctuations in the microscopic degrees of freedom, converting them into random bits. Looking only at the central bistable system, the random sequence thus generated amounts to a production of one bit of entropy per run of the experiment. Here, another aspect of the Hamiltonian dynamics of closed systems comes in handy, the conservation of entropy under canonical transformations [16]. It implies that the entropy in the random sequence cannot be produced by the central system but must originate somewhere else in the total system. The only possible source is the environment embodied in the heat bath. From a different point of view, the falling pencil violates the rotational symmetry with respect to the vertical axis of the total potential, including the interaction with the environment, and of its own initial condition. The symmetry breaking must therefore occur in the initial condition of the environment. Finally, with this random bit the system retains a lasting memory, albeit minimal, of its initial state, a blatant manifestation of its non-Markovian nature.

Deterministic chaos reduces entropy production to the expansion of the initial condition by the chaotic phase-space flow [17]. To be sure, already for N=1, the quartic double well coupled to harmonic oscillators is indeed a partially chaotic system. However, this is not decisive for the randomness exhibited by the bistable system. The pivotal factor is rather the many-body nature of the bath. In this sense, what we see is Brownian motion discretized and condensed into random bits. Another surprising instance of macroscopic randomness generated by a microscopic dynamics, amenable to a deterministic description, is diffusion-limited deposition, modelled as and simulated by lattice-gas automata [18].

In fact, this work is inspired and motivated by a similar situation in quantum mechanics. Spin measurement is a paradigm of irreducible randomness in quantum mechanics, it serves as a source of binary random numbers less predictable than any classical physical or digital random number generator, and therefore a valuable resource and a gold standard for applications such as cryptography [19,20,21]. A quantum two-state system such as a spin-$\frac{1}{2}$ neither has a classical limit nor can it be understood as the quantization of a classical bistable system. However, the double-well potential is regarded as the closest classical analogue of a qubit, and the isolated ground-state pair of a quantum double well can be mapped one-to-one to a qubit [22]. Inhowfar the results of the present work suggest any new insight concerning the interpretation of quantum randomness is presently under study.

We review the anatomy of the quartic double well in Section 2.1, together with an outline of the Hamiltonian as well as the dissipative dynamics of this bistable system. Section 2.2 details the construction of the heat bath and sketches some basic facts about the irreversible relaxation process approached in the limit N→∞ of the number *N* of bath modes. Numerical results confirming and illustrating the chaotic behaviour of the double well coupled to a single harmonic oscillator, N=1, are presented in Section 3. The central Section 4 is dedicated to our main results providing numerical evidence for the relaxation of the bistable system into one of its stable equilibrium positions for N≫1 and the dependence of the final state on the initial condition of the bath. Finally, Section 5 reflects on the implications of our results for our conception of randomness.

## 2. The Model: Bistable System Coupled to a Finite Heat Bath

A straightforward way of modelling a multistable system is combining a potential with a corresponding number *n* of relative minima with a dissipative dynamics, for example Ohmic friction. In the overdamped regime, where inertia can be neglected against potential forces and friction, the system will fall from any initial condition into the closest well. We here follow this simple scheme to model a bistable system, that is, for n=2, to be construed in Section 2.1, before coupling it to a finite heat bath in Section 2.2.

### 2.1. Quartic Double Well

Modelling a bistable system, any potential with two symmetry-related minima will do, but for the sake of mathematical transparency and ease of calculation, we prefer the standard potential of a quartic oscillator with a parabolic barrier (Figure 1a),

(1) $$V_{S}\left(X\right)=-\frac{a}{2}X^{2}+\frac{b}{4}X^{4},\;a,b\in R^{+}.$$

It has quadratic minima at $X_{\pm }=\pm \sqrt{a/b}$ and a quadratic maximum at $X_{0}=0$ of relative height $E_{B}=a^{2}/4b$. The only relevant parameter for the shape of this potential is the relative sign of *a* and *b*, even a variation of the ratio a/b can be compensated by a corresponding rescaling of position or energy or both.

Frictionless motion in this potential (Figure 1b) is described by the Hamiltonian

(2) $$H\left(P,X\right)=\frac{P^{2}}{2M}+V\left(X\right).$$

Close to the minima at $X_{\pm }$, it consists of harmonic oscillations with the frequency $\Omega =\sqrt{2a/M}$. They become increasingly anharmonic as the energy rises towards the top of the barrier. At E=0, on the level of the barrier top, the trajectory assumes a figure-eight shape, the separatrix (red curve in Figure 1b). Close to the top, the dynamics is governed by an unstable manifold, along which distances in phase space increasing exponentially with the Lyapunov exponent $\Lambda =\sqrt{a/M}$, and a stable manifold contracting phase space correspondingly. At higher energies E>0, oscillations are strongly anharmonic and circle both wells, passing over the barrier back and forth.

Remaining on the macroscopic level of description, dissipation is included as a damping term in Newton’s equations of motion, as derived otherwise from the Hamiltonian (2),

(3) $$M\ddot{X}=aX-bX^{3}-2\Gamma \dot{X}.$$

Assuming Ohmic friction with damping coefficient 2Γ. Solutions of Equation (3) now contract phase space exponentially with a rate 2Γ towards a pair of point attractors, one at the bottom of each minimum. In the underdamped regime, for Γ<Ω, from an initial energy E(0)<0, below the top of the barrier, trajectories spiral from either side of the barrier into the adjacent well, the basins of attraction forming a Yin-and-Yang figure (Figure 1c). At higher energies, they wind out around one another and around the two wells. In the overdamped regime, these spirals get steeper, the basins of attraction approaching the half spaces $X\in R^{-}$ for $X_{-}$ and $X\in R^{+}$ for $X_{+}$, resp (Figure 1d).

In all regimes, independently of the degree of friction, the equations of motion are solved by (P,X)=const=(0,0) if the system is prepared at rest on top of the barrier. This is an isolated point in phase space. With nonzero friction, any infinitesimal deviation from this unstable equilibrium will send the system into one of the wells. Even in the Hamiltonian case, trajectories started on the separatrix do not pass through this point but need an infinite time to approach it.

### 2.2. Finite Heat Bath

Coupling the bistable system to an environment with a large number of degrees of freedom requires including three terms in the Hamiltonian,

(4) $$H\left(R,r\right)=H_{S}\left(R\right)+H_{SE}\left(R,r\right)+H_{E}\left(r\right),$$

R=(P,X) denoting the phase-space coordinates of the central system and $r=(p_{1},p_{2},\ldots ,p_{N},$ $x_{1},x_{2},\ldots ,x_{N})$ those of the environment comprising *N* degrees of freedom. The self-energy of the central system is given by the quartic double well, Hamiltonian (2). For the environment we choose a set of *N* harmonic oscillators,

(5) $$H_{E}\left(r\right)=\sum_{n=1}^{N}\left(\frac{p_{n}^{2}}{2m}+\frac{m\omega_{n}^{2}}{2}x_{n}^{2}\right).$$

**Figure 2:**
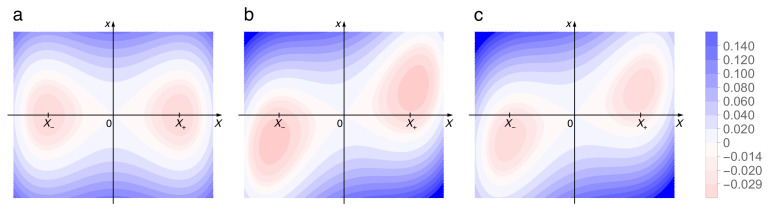
Contour plots of the potential (15) with parameters a=b=0.15, m=1, ω=0.4, without coupling (**a**), with coupling g=0.06, but not including the counter term $g^{2}X^{2}/\left(2m\omega^{2}\right)$ (**b**), and with this term (**c**). Colour code ranges from red (negative) through white (zero) through blue (positive).

The frequencies $\omega_{n}$, n=1,…,N, will be specified further below. Every oscillator should exert a force, constant in space, on the central system. This suggests modelling their interaction as a linear position-position coupling,

(6) $$H_{SE}\left(R,r\right)=H_{SE}\left(X,x\right)=-X\sum_{n=1}^{N}g_{n}x_{n},$$

with coupling constants $g_{n}$, n=1,…,N. It does not break the invariance of the total system under parity (spatial reflection) P: (r,R)→(−r,−R). However, it drives the two minima apart, from $X_{\pm }=\pm \sqrt{a/b}$ to $X_{\pm }=\pm \sqrt{\frac{1}{b}\left(a+\sum_{n=1}^{N}g_{n}^{2}/2m\omega_{n}^{2}\right)}$, see Figure 2b, an effect not intended with the coupling to an environment. It can be compensated for by including a counter term $\sim X^{2}$ in the potential to complete the squares with respect to the dependence on the oscillator coordinates, see Figure 2c,

(7) $$\begin{array}{cc} V_{SE}\left(X,x\right) & =V_{S}\left(X\right)+\sum_{n=1}^{N}\frac{m\omega_{n}^{2}}{2}x_{n}^{2}-X\sum_{n=1}^{N}g_{n}x_{n}+X^{2}\sum_{n=1}^{N}\frac{g_{n}^{2}}{2m\omega_{n}^{2}}\\ \; & =V_{S}\left(X\right)+\sum_{n=1}^{N}\frac{m\omega_{n}^{2}}{2}{\left(x_{n}-\frac{g_{n}}{m\omega_{n}^{2}}X\right)}^{2}. \end{array}$$

Without pretending any kind of rigorous quantization, we consider the Hamilton operator

(8) $$\hat{H}={\hat{H}}_{S}+{\hat{H}}_{SE}+{\hat{H}}_{E},\;{\hat{H}}_{S}=\frac{1}{2}\hbar \Omega {\hat{\sigma }}_{x},\;{\hat{H}}_{SE}=\sum_{n=1}^{N}{\hat{\sigma }}_{z}\left(g_{n}{\hat{a}}_{n}^{†}+g_{n}^{*}{\hat{a}}_{n}\right),\;{\hat{H}}_{E}=\sum_{n=1}^{N}\hbar \omega_{n}\left({\hat{a}}_{n}^{†}{\hat{a}}_{n}+\frac{1}{2}\right),$$

with Pauli spin matrices, ${\hat{\sigma }}_{x}$ and ${\hat{\sigma }}_{z}$, and boson creation and annihilation operators, ${\hat{a}}_{n}^{†}$ and ${\hat{a}}_{n}$, resp., known as *spin-boson model* [22,23,24,25], as a close quantum analogue of the classical Hamiltonian (4)–(7).

In the limit N→∞ of a quasicontinuous spectrum of the bath oscillators and under certain conditions on the spectral density and the frequency dependence of the coupling, the dynamics of the central system exhibits irreversible relaxation into a stable state, superposed with stochastic fluctuations. To be more precise, a decisive quantity is the coupling strength function [7], defined by

(9) γ(ω)dω:=∑nω≤ωn≤ω+dωgn2.

Close to either one of the quadratic minima of the double well, if the total coupling is not too strong,

(10) $$G^{2}:=\int_{0}^{\infty }d\omega \frac{\gamma (\omega )}{\omega^{2}}\leq \Omega^{2}.$$

The central system behaves as a harmonic oscillator subject to Ohmic friction and a fluctuating force. The equations of motion for X(t) in general take the form of integro-differential equations with integral kernels that are nonlocal in time [7]. However, if $\omega^{-2}\gamma \left(\omega \right)$ is approximately constant within a frequency range Δω containing Ω, the autocorrelation time of the bath responsible for the memory, reduces as $\tau_{E}\sim \Delta \omega^{-1}$, and for Δω≫Ω, the response of the bath decays instantaneously. For much larger frequencies, γ(ω) must be cut off, say exponentially $\gamma \left(\omega \right)\sim \exp\left(-\omega /\omega_{co}\right)$, in order to satisfy Equation (10).

Under these assumptions, the dynamics of the central system is described by a Langevin equation [5,6,7,26],

(11) $$\ddot{X}\left(t\right)+2\Gamma \dot{X}\left(t\right)+\Omega^{\prime 2}X\left(t\right)=\frac{1}{M}\tilde{F}\left(t\right),$$

with a friction coefficient $\Gamma =\pi \gamma \left(\Omega \right)/4\Omega^{2}$ and a modified frequency $\Omega^{\prime }=\sqrt{\Omega^{2}-\Gamma^{2}}$. The fluctuating force $\tilde{F}\left(t\right)$ with zero mean, $\langle \tilde{F}\left(t\right)\rangle =0$, is delta-correlated,

(12) $$\langle \tilde{F}\left(t\right)\tilde{F}\left(t+s\right)\rangle \sim \delta \left(s\right).$$

In the vicinity of the barrier top, similar considerations apply, but with the natural frequency $\Omega =\sqrt{2a/M}$ of oscillations in the wells replaced by the Lyapunov exponent associated to the parabolic barrier, taken as an imaginary frequency, $\Omega \to i\Lambda =i\sqrt{a/M}$. The Langevin equation analogous to Equation (11), valid in this neighbourhood, therefore reads

(13) $$\ddot{X}\left(t\right)+2\Gamma \dot{X}\left(t\right)-\Lambda^{\prime 2}X\left(t\right)=\frac{1}{M}\tilde{F}\left(t\right),$$

and is solved by trajectories expanding or contracting with the rates $-\Gamma \pm \Lambda^{\prime }$, $\Lambda^{\prime }=\sqrt{\Lambda^{2}+\Gamma^{2}}$, along the unstable and stable manifolds in phase space, resp., emanating from the top of the barrier. In terms of the interplay of Equation (13), valid near the maximum, and Equation (11), valid near the minima, it is the initial amplification of the fluctuating force $\tilde{F}\left(t\right)$ along the unstable manifold, frozen in and reduced to either one of two asymptotic states for $t\gg \Gamma^{-1}$, that interests us here. Details of the way the bistable system coupled to a finite bath approaches these states will be discussed in Section 4.2 and Section 4.3.

## 3. Double Well Coupled to a Single Harmonic Oscillator or a Few of Them: Chaotic Dynamics

An important aspect of our model, to be contrasted with the regime N≫1 of a bath comprising a large number of degrees of freedom, is the case N=1 of a quartic double well coupled to a single harmonic oscillator. With its two degrees of freedom, it is still far from even any symptoms of relaxation. However, involving strong anharmonicity in one of its freedoms, it meets all conditions to become chaotic for non-zero coupling. In this section, we present numerical evidence that this is indeed the case.

The total Hamiltonian for N=1 reads,

(14) $$H\left(R,r\right)=\frac{P^{2}}{2M}+\frac{p^{2}}{2m}+V_{SE}\left(X,x\right),$$

where (Figure 2)

(15) $$V_{SE}\left(X,x\right)=-\frac{a}{2}X^{2}+\frac{b}{4}X^{4}+\frac{m\omega^{2}}{2}x^{2}-gxX+X^{2}\frac{g^{2}}{2m\omega^{2}}=-\frac{a}{2}X^{2}+\frac{b}{4}X^{4}+\frac{m\omega^{2}}{2}{\left(x-\frac{g}{m\omega^{2}}X\right)}^{2}.$$

It is invariant under parity, (P,X,p,x)→(−P,−X,−p,−x), but to our best knowledge lacks any other symmetry or constant of motion. At the same time, both subsystems are separately integrable for g=0, so that we expect to see a generic Kolmogorov-Arnol’d-Moser (KAM) [27] scenario in the transition from purely regular to strongly chaotic motion with increased.

In all regimes, independently of the degree of friction, a solution of the equations of motion is (P,X)=const=(0,0) if the system is prepared in this state on top of the barrier. This is an isolated point in phase space. With nonzero friction, any infinitesimal deviation from this unstable equilibrium will send the system into one of the wells. In the Hamiltonian case, even trajectories started on the separatrix never reach this point but need an infinite time to approach it.

Numerical solutions of Hamilton’s equations of motion with the Hamiltonian (14) and (15) have been obtained with a symplectic integration routine based on a first-order Verlet Leapfrog algorithm [28,29,30]. Figure 2a shows contour lines of the potential $V_{SE}\left(X,x\right)$, without coupling (Figure 2a) and for g=0.06, without counter term, see Equation (7) (Figure 2b) and with it (Figure 2c). Note that without the counter term, the projections onto *X* of the positions of the two minima get shifted towards larger values of |X| (panel (b)), but return to their original values $X_{\pm }$ if it is included (panel (c)). The parity or, equivalently in two dimensions, $C_{2}$-symmetry is evident.

**Figure 3:**
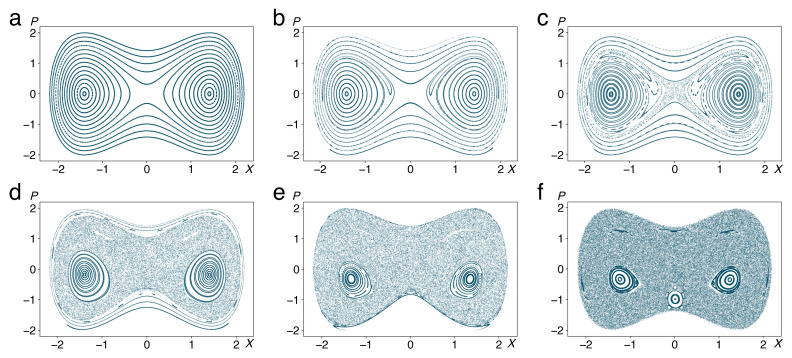
Poincaré surfaces of section for the motion generated by the Hamiltonian (14) and (15), showing intercepts (P,X) with the hyperplane x=0 under the condition p>0, for g=0 (**a**), g=0.001 (**b**), g=0.005 (**c**), g=0.05 (**d**), g=0.1 (**e**), g=0.2 (**f**). Other parameters are a=2, b=1, M=1, m=0.1, ω=1.5.

In order to visualize trajectories of the system, we use Poincaré surfaces of section [27,31] to reduce the three dimensions of the energy shell, the invariant manifold containing the trajectories within the four-dimensional phase space, further to two. Coordinates $\left(P(t),X(t)\right)$ are registered whenever trajectories intersect the plane x=0 with p>0 at times $t_{n}$ (not necessarily equidistant), generating discrete point sequences $\left(P_{n},X_{n}\right)$. Surfaces of section for different values of the coupling constant *g* are presented in (Figure 3). For g=0 (Figure 3a), point patterns follow one-dimensional curves that coincide with the contours of the potential, Figure 2a. Increasing the coupling to g=0.001 (b) and further to g=0.005 (c), we see irregular motion invading phase space in the vicinity of the separatrix, in the form of chains of regular islands surrounded by chaotic regions. The total phase-space area occupied by chaotic trajectories expands further into the wells and the region above the barrier, resembling a Venetian half mask, as *g* increases to 0.05 (d) and 0.1 (e), till the remaining regular regions reduce to small islands around the bottoms of the two wells, at g=0.2 (f). The small island visible at the “mouth of the mask”, near (P,X)=(−1,0), pertains to a stable periodic trajectory that follows roughly one of the contours of the potential, see Figure 2, at V(X,x)>0, and represents a surprising effect of the strongly nonlinear dynamics. With this behaviour, the system follows the well-known KAM scenario.

**Figure 4:**
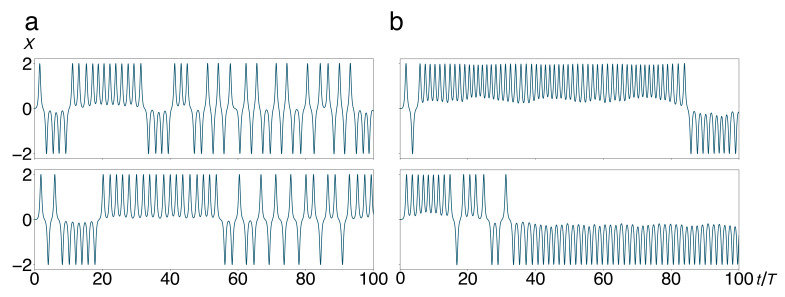
Trajectories X(t) of the quartic double well coupled to a small number *N* of harmonic oscillators, Equations (1), (2), and (4)–(7), launched from an initial state at rest on top of the barrier, $\left(P(0),X(0)\right)=\left(0,0\right)$, show sporadic transitions (“jumps”) between the wells, for N=1 (**a**) and N=5 (**b**). Sample trajectories shown in the upper panels differ from those in the lower ones only by the initial conditions of the degree(s) of freedom of the harmonic oscillator(s). Time is measured in units of the period T=2π/Ω of unperturbed oscillations around the stable equilibria of the double well. Other parameter values are M=1, m=1, g=0.1 and ω=1.5. For N=5, frequencies and initial conditions of the bath oscillators have been drawn from random distributions, see Section 4.1.

In Section 4.3, we shall focus on the random nature of the decision whether the system, coupled to a larger number of harmonic oscillators, will decay into the left or the right well, thus generating a binary random sequence of symbols, say 0 s and 1 s. Associating these symbols to the two half spaces {x|x<0} and {x|x≥0}, we could already describe the trajectories of the double well coupled to a single bath mode, depicted in Figure 4, as discrete symbol sequences. Interpreting the two half spaces as a Markov partition, these sequences could be amenable to an analysis in terms of symbolic dynamics [17,27,32].

With more oscillator modes added to the environment, the total system does of course not return to integrability. Yet, in a different sense, the dynamical disorder does reduce: The frequency of jumps between the two wells (analogous to spin flips in the quantum mechanical context) diminishes with increasing *N*. In Figure 4, we depict sample trajectories, representing the position X(t) of the central system alone, for N=1 (a) and 5 (b). We observe a tendency that the frequency of jumps decreases and the duration of localized episodes, that is, periods where the system remains in one of the two wells, increases. It can be roughly explained by the fact that an increasing fraction of the total energy of the system is absorbed by the bath oscillators, so that most of the time, the central system does not have enough energy to surmount the barrier.

## 4. Double Well Coupled to a Large Bath: Relaxation and Localization at Random

Increasing *N* further, we approach the regime where it is more appropriate to treat the bath modes statistically. Keeping their number finite, though, we are invariably dealing with discrete distributions of frequencies, coupling strengths, and so forth, and thus have to be more specific than working with ensembles defined completely by smooth probability densities. In the incipient field of finite baths, a few strategies, mostly for the context of quantum systems, have been developed to cope with this situation [33], some of which we adopt in the present work.

### 4.1. General Setup of Numerical Simulations

Basic data on the dynamics of a quartic double well coupled to an environment comprising *N* harmonic oscillators, defined by Equations (1), (2), and (4)–(7), are gained by solving Hamilton’s equations of motion,

(16) $$\begin{array}{cc} \dot{P} & =aX-bX^{3}+\sum_{n=1}^{N}g_{n}x_{n},\;\dot{X}=\frac{P}{M},\\ {\dot{p}}_{n} & =-m\omega_{n}^{2}x+g_{n}X,\;{\dot{x}}_{n}=\frac{p_{n}}{m},\;n=1,\ldots ,N, \end{array}$$

using a Calvo-Sanz-Serna $4^{\mathrm{th}}$ order symplectic integrator [28,29,30]. A guideline for the definition of frequencies $\omega_{n}$ and couplings $g_{n}$ are the conditions, mentioned in Section 2.2, for Ohmic friction and a delta-correlated fluctuating force. They suggest to choose the coupling strength function (9) as

(17) $$\gamma \left(\omega \right)=\sum_{n=1}^{N}g_{n}^{2}\delta \left(\omega -\omega_{n}\right)\sim \omega^{2}\exp\left(-\frac{\omega }{\omega_{co}}\right).$$

Including an exponential cutoff at $\omega_{co}$. In the context of quantum decoherence and dissipation, the relevant quantity considered instead of γ(ω) is the so-called *spectral function* [7,22,33],

(18) $$J\left(\omega \right):=\frac{\pi }{2}\sum_{n}\frac{g_{n}^{2}}{m\omega_{n}}\;\delta \left(\omega -\omega_{n}\right).$$

Writing the spectral function as a product

(19) $$J\left(\omega \right)=f\left(g(\omega )\right)\rho_{\omega }\left(\omega \right),$$

makes it explicit that it combines the effects of the frequency dependence $f\left(g(\omega )\right)$ of the coupling with the pure density of states $\rho_{\omega }\left(\omega \right)$. For this product, the frequency dependence equivalent to Equation (17) is

(20) $$J\left(\omega \right)\sim \omega \exp\left(-\frac{\omega }{\omega_{co}}\right).$$

**Figure A1:**
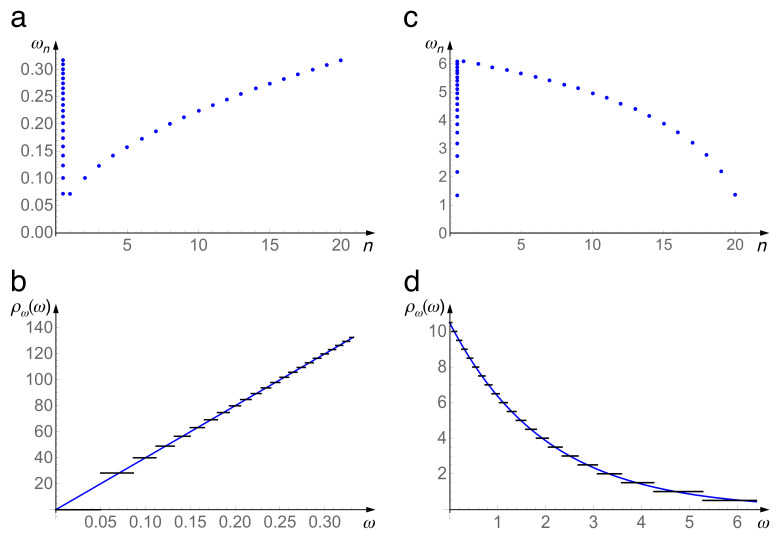
Results of the spectral discretization procedure detailed in Appendix A, for an Ohmic spectral density without cutoff, Equation (A14) (panels (**a**,**b**)), and a pure exponential cutoff, Equation (A15) (**c**,**d**). Top (**a**,**c**): discrete frequencies $\omega_{n}$ vs. level index *n*, projected onto a line near the vertical axis to visualize the density. Bottom (**b**,**d**): Target spectral density (full blue curves) compared to the inverse step size (black stairs), Equation (A6), generated by the procedure.

We assemble sets of *N* harmonic oscillators that satisfy Equation (17) by adapting only the density of states to this condition while keeping the couplings constant within the bath for given *N*. In order to maintain the total interaction energy independent of the number of degrees of freedom in the bath, we scale the couplings globally with *N* as

(21) $$g_{n}=const=\frac{g}{\sqrt{N}},\;n=1,\ldots ,N,$$

with a global system-bath coupling *g*. At the same time, we define a sequence of discrete frequencies $\omega_{n}$, not equidistant but with variable frequency steps adjusted such that the resulting spectral density satisfies Equation (17), see Appendix A. For an Ohmic coupling strength function $\gamma \left(\omega \right)\sim \omega^{2}$ for $\omega \ll \omega_{co}$, this implies to discretize the frequencies as (Figure A1a)

(22) $$\omega_{n}=\Delta \omega \sqrt{2n},\;\omega \ll \omega_{co}.$$

The exponential cutoff on a frequency scale $\omega_{co}$ included in Equation (17) is achieved by a discretization (Figure A1c)

(23) $$\omega_{n}=-\omega_{co}\ln\left(N_{co}-n\right),\;\omega \gg \omega_{co},$$

choosing the parameter $N_{co}$ according to the desired maximum frequency $\omega_{N}=-\omega_{co}\ln\left(N_{co}-N\right)$.

The initial state of the environment playing a central role for our reasoning, we have to treat the initial conditions in phase space of the *N* harmonic oscillators with particular care. We consider both position $x_{n}$ and momentum $p_{n}$ of each oscillator as Gaussian random variables, defined by probability density functions

(24) $$\rho_{p}\left(p_{n}\right)=\frac{1}{\sqrt{\pi E_{p}}}\exp\left(-\frac{1}{E_{p}}\frac{p_{n}^{2}}{2m}\right),\;\rho_{x}\left(x_{n}\right)=\frac{1}{\sqrt{\pi E_{x}}}\exp\left(-\frac{1}{E_{x}}\frac{m\omega_{n}^{2}}{2}x_{n}^{2}\right).$$

Independent variances $E_{p}$ and $E_{x}$ for momentum and position preserves us the freedom to vary the aspect ratio of the resulting Gaussian clouds in phase space, but in most cases, we fix the widths such that $E_{p}=E_{x}=:E_{ho}$. The total initial energy in the bath will be kept constant,

(25) $$E_{bath}=\sum_{n=1}^{N}E_{n},$$

mostly at a fraction of the barrier height, so that the individual initial energies scale on average as $E_{n}\sim E_{bath}/N$. In order that they comply with Equation (25) exactly, the $E_{n}$ are adapted to the required value of $E_{bath}$ by scaling all initial positions and momenta accordingly by the same factor $\sqrt{E_{bath}/\sum_{n=1}^{N}E_{n}}$.

It is tempting to interpret the densities (24) as Boltzmann distributions, defining a temperature through $E_{ho}=k_{B}T$. However, in view of the wider scope of this work towards randomness of any origin, we avoid a narrow interpretation in thermodynamical terms and consider Gaussian distributions as in Equation (24) as a practical, rather than compelling, choice.

### 4.2. Relaxation to Stationary States

In order to demonstrate that the double well coupled to a finite environment, for sufficiently large values of *N*, does approach states that are nearly stationary over long timescales, we refer to different diagnostics of irreversible behaviour, some of more local, some of more global character. An appropriate indicator of the loss of memory is the autocorrelation as a function of the time shift [6,7]. For the position of the central system, it is defined as

(26) $$\chi_{XX}\left(t,t+s\right)=\frac{\langle \left(X(t)-\langle X\rangle \right)\left(X(t+s)-\langle X\rangle \right)\rangle }{\langle X^{2}\rangle }.$$

The angle brackets denote averaging over an ensemble of baths as indicated above. Moreover, for each configuration of the bath, after transients have decayed, the process can be considered stationary and we can also average over time *t* in each time series, keeping the time shift *s* constant.

**Figure 5:**
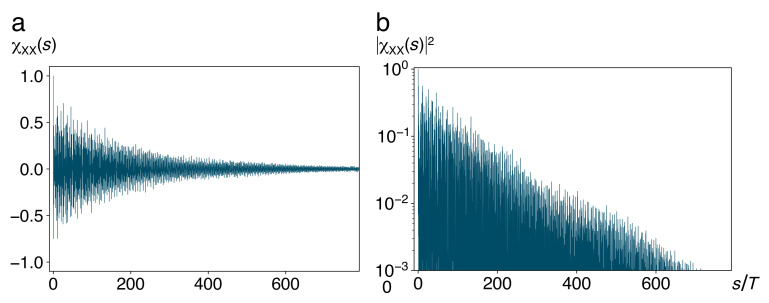
Autocorrelation $\chi_{XX}\left(s\right)$, cf. Equation (26), of the position X(t) of the central system (**a**). Despite averaging over ensembles of initial conditions of the bath as well as the absolute time in times series of X(t), oscillations with the frequency Ω of harmonic motion around the stable equilibria survive longer than bath oscillations proper. A semilogarithmic plot of the square ${\left|\chi_{XX}\left(s\right)\right|}^{2}$ (**b**) confirms their exponential decay. Time axes in units of T=2π/Ω as in Figure 4. Other parameter values are a=2, b=1, g=0.5, $E_{bath}=0.1$, M=1, m=0.1, N=15, $\omega_{co}=4$.

An example of the time-averaged autocorrelation $\chi_{XX}\left(s\right)$ is plotted in Figure 5a. Superposed on the long-term exponential decay of the envelope, we observe rapid oscillations of the autocorrelation, with the frequency Ω of harmonic motion around the stable equilibria on the bottom of each well. They decay much slower than the fluctuations of the heat bath. In panel (b), we show a semilogarithmic of the square ${\left|\chi_{XX}\left(s\right)\right|}^{2}$, circumventing negative values of $\chi_{XX}\left(s\right)$, as direct evidence of the exponential decay of the autocorrelation.

The process of relaxation of the central system towards a stationary state in one of the wells should be reflected in a characteristic time dependence of the entropy of the subsystems. While the total entropy of the system double well plus environment is conserved under canonical transformations, the sum of partial entropies of subsystems may vary. In terms of the reduced probability density of the double well,

(27) $$\rho_{S}\left(R,t\right)=\int d^{2N}r\;\rho \left(R,r\right)=\int dp_{N}\int dx_{N}\cdots \int d\;p_{1}\int dx_{1}\;\rho \left(R,r,t\right).$$

The partial entropy in this subsystem is given by [2,34]

(28) $$S_{S}=-c\int d^{2}R\;\rho_{S}\left(R\right)\ln\left(\rho_{S}\left(R\right)\Delta A\right),$$

where ΔA is the symplectic area of a minimal phase-space cell resolved by the input data, and we choose c=1/ln(2), measuring entropy in units of bits. We evaluate the entropy by launching a set of *K* trajectories, initially concentrated within a single bin ΔA=ΔPΔX of a discretized phase space, and counting the number of trajectories found at time *t* in each bin at $\left(P_{\lambda },X_{\mu }\right)$, λ=1,…,L, μ=1,…,M, to determine probabilities $p_{\lambda ,\mu }\left(t\right)$. The entropy is then calculated as

(29) $$S_{S}\left(t\right)=-c\sum_{\lambda =1}^{L}\sum_{\mu =1}^{M}p_{\lambda ,\mu }\left(t\right)\ln\left(p_{\lambda ,\mu }\left(t\right)\right).$$

**Figure 6:**
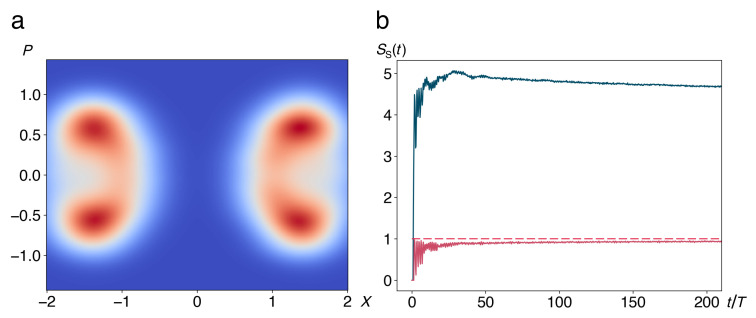
(**a**) Snapshot of the reduced density $\rho_{S}\left(P,X,t\right)$, Equation (27), at t=200T, T=2π/Ω, featuring the bimodal distribution with one peak in each of the two minima of the double well. Colour code ranges from zero (blue) through moderate (white) through high positive values (red). (**b**) Time evolution of the partial entropy $S_{S}\left(t\right)$ of the central system, Equation (29), initially prepared at rest on top of the barrier, calculated with different degrees of resolution of phase space. For a large bin size ΔA=3 (red), only the splitting of the asymptotic distribution into two peaks is resolved, resulting in an asymptotic entropy $S_{S}\left(t\right)\to 1bit$ (dashed horizontal line). For ΔA=0.5 (blue), the entropy is dominated by the finite width of the two peaks, approaching an asymptote $\lim_{t\to \infty }S_{S}\left(t\right)\gg 1bit$. Time axis in units of *T* as in Figure 4. Other parameter values are a=2, b=1, g=0.1, $E_{bath}=0.1$, M=1, m=0.1, N=15, $\omega_{co}=4$.

By construction, $S_{S}\left(0\right)=0$. Time series of the partial entropy in the degree of freedom of the double well are presented in Figure 6. If the phase-space resolution ΔA is chosen comparable to the area of the two maxima of the bimodal asymptotic density distribution (Figure 6a), the entropy approaches an asymptote of 1 bit (red curve and dashed horizontal line in Figure 6b). For a higher resolution (blue curve in Figure 6b), the major part of the entropy is contributed by the nonzero widths of the two peaks, leading to a value far above 1 bit. With increasing resolution, the number of bins covered by the asymptotic distribution grows, and so does accordingly the long-time limit of the entropy.

Calculating the partial entropy of the environment, to compare it with that of the central system, requires discretizing a 2N-dimensional phase space into reasonably fine bins, a task that is unfeasible with the computer equipment available to us.

**Figure 7:**
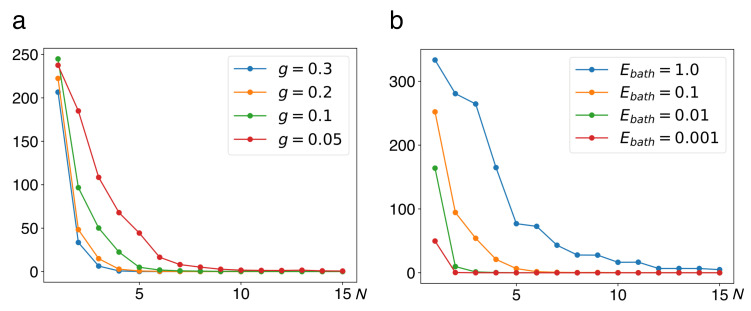
Frequency of jumps of the central system as a function of the number *N* of bath modes, varying the global coupling *g* (**a**) and the total energy in the bath $E_{bath}$ (**b**). The number of jumps occurring within 100 periods T=2π/Ω of unperturbed oscillations around the stable equilibria of the double well has been averaged over ensembles of 100 initial conditions of the heat bath oscillators. Other parameter values are a=2, b=1, $E_{bath}=0.1$, M=1, m=0.1, $\omega_{co}=4$.

Finally, a simple direct criterion for the relaxation of a bistable system to a stable equilibrium is the frequency of jumps between the two wells. They require a kinetic energy of the order of the barrier height to be concentrated in the central degree of freedom, that is, an exceptionally strong fluctuation. Therefore they become less and less likely as the number of oscillators in the environment increases. We present evidence for this tendency in Figure 7, plotting the number of jumps, accumulated over a constant measurement period Δt=100T, with T=2π/Ω, as a function of the number *N* of modes in the heat bath, varying the global coupling *g*, cf. Equation (21) (Figure 7a) and the total energy in the bath $E_{bath}$, cf. Equation (25) (Figure 7b). As is to be expected, the sojourn time in either minimum grows with increasing coupling strength and with decreasing energy in the bath.

### 4.3. Amplified Fluctuations: Randomness in the Approach to an Asymptotic State

Even if the time from the initial relaxation into one of the two minima till the next jump to the other side and between subsequent jumps diverges with increasing size of the bath, the Poincaré recurrence theorem [15] implies that the system will return infinitely often to a state within an ϵ-neighbourhood of its initial state (on top of the barrier) for every $\epsilon \in R^{+}$. In the present context, such a near recurrence occurs every time the system passes over the barrier, moving from one well to the other. In this sense, there is no such thing as a “final state” of a double well coupled to a finite heat bath. Notwithstanding, the time from one jump to the next, thus between two subsequent recurrences, rapidly exceeds every physically relevant timescale. Moreover, in the same guise of a realistic modelling of a system embedded in its environment, with increasing time, weaker couplings to remoter systems, hence larger environments, have to be taken into account, leading to a further stabilization of the state the system had relaxed to for the first time. These arguments justify talking of a “final state” in a heuristic sense.

**Figure 8:**
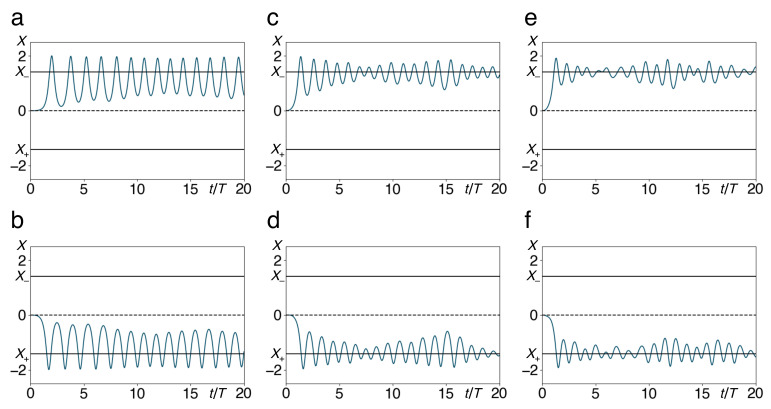
Sample trajectories of the quartic double well coupled to *N* harmonic oscillators, Equations (4)–(7), falling from an initial state at rest on top of the barrier, $\left(P(0),X(0)\right)=\left(0,0\right)$, into the left or the right well, plotted as traces position vs. time. Time axes in units of T=2π/Ω as in Figure 4. The stable equilibria $X=X_{\pm }$ and the unstable equilibrium at X=0 of the double well are marked by full and dashed horizontal lines, resp. Parameter values are g=0.1 (**a**,**b**), 0.3 (**c**,**d**), 0.5 (**e**,**f**), and a=2, b=1, $E_{bath}=0.1$, M=1, m=0.1, $\omega_{co}=4$, N=15. Frequencies and initial conditions of the bath oscillators have been drawn from random distributions, see text. Trajectories shown in the upper differ from those in the lower panels only by these random parameters.

In Figure 8, we present a few example trajectories which, starting from a state at rest at the top of the barrier, eventually fall into one of the two wells and then merely fluctuate around the corresponding minimum. As the statistics of jumps in the foregoing subsection already indicates, the amplitude of these fluctuations diminishes with increasing size of the heat bath and with the coupling to it, but increases with the total energy in the bath, that is, in a thermodynamical context, with increasing temperature. These trajectories are reproducible—prepared in the same initial states of the double well and in particular of the bath oscillators, the system approaches the same quasi-stationary state in the same way.

**Figure 9:**
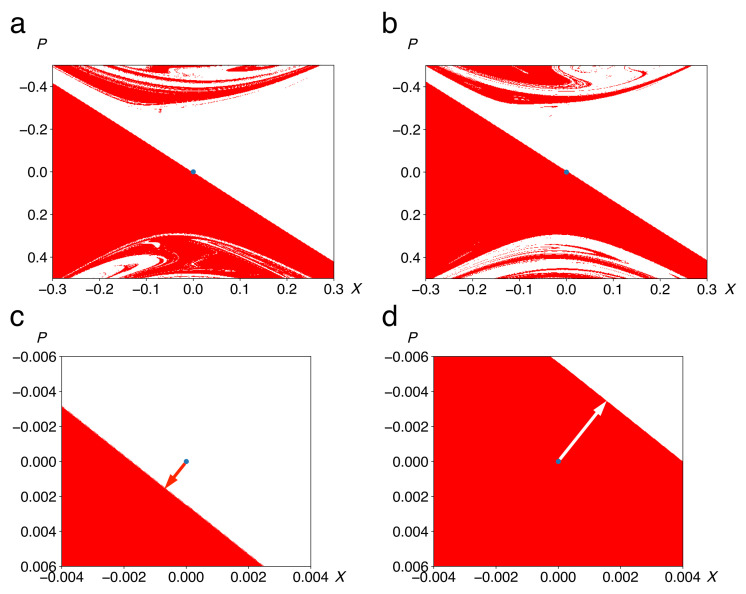
Basins of attraction of the two stable equilibria of the double well as in Figure 1c, but for different specific initial conditions of the bath. Colours (red vs. white) indicate the side the system approaches in its initial relaxation. Panels (**a**) and (**b**) are total views of the central part of the basins, showing their disturbance by the initial condition of the bath. Close-ups (**c**,**d**) demonstrate the shift of the boundary (arrows), away from the origin $\left(P(0),X(0)\right)=\left(0,0\right)$ (blue dot), if the bias imparted by the bath lets the system fall from the top of the barrier into the right (**c**) or the left well (**d**). Parameter values are a=2, b=1, g=0.1, $E_{bath}=0.1$, M=1, m=0.1, N=15, $\omega_{co}=4$.

Figure 9 provides evidence for this scenario from a different point of view. We depict the basins of attraction of the two wells (left well red, right well white) in the phase space of the central system, now defined by the side the system approaches in its first relaxation, *keeping the initial condition of the bath fixed*. That means that the boundary no longer passes exactly through the top of the barrier, as it does for the Newtonian equation of motion with dissipation, Equation (3), see Figure 1c. If for a given initial state of the bath, the central system falls from the top of the barrier into, say, the left well, this implies that an opposite bias has to be imposed on the initial condition of the central system, for example an initial position slightly to the right of the barrier top or a small positive momentum, to compensate for the initial bias of the bath. In this way, the boundary is shifted towards positive position or momentum, into the first quadrant of the phase space of central system (white arrow in Figure 9d), and vice versa if from $\left(P(0),X(0)\right)=\left(0,0\right)$, it falls into the right well (red arrow in Figure 9c). Direction and magnitude of these shifts depend on the initial condition of the bath, chosen at random. On average over a sufficiently large ensemble of initial states, they cancel, reflecting the condition $\langle \tilde{F}\left(t\right)\rangle =0$, cf. Equation (11).

Panels (a) and (b) of Figure 9 show the shape of the basins within a relatively large phase-space domain. The self-similar patterns superposed on the smooth Yin-and-Yang shape of Figure 1c reflect the nonlinear nature of the dynamics as well as the random character of the initial state of the bath. These structures resemble fractal basin boundaries, a characteristic of dissipative chaotic systems with two or more coexisting attractors [35,36]. Panels (c) and (d) show close-up views of the basin boundary, close to the origin (P,X)=(0,0), for two different initial conditions of the bath. The shift of away from the origin is obvious.

## 5. Conclusions

With the project presented in this report, we have explored new ground in several respects. By contrast to the theory of deterministic chaos, we here study the origin of randomness in *discrete* time series, such as those generated by games of luck, in a deterministic dynamics. We substantiate our approach by constructing a detailed model of a bistable system interacting with a many-body environment, a quartic double well coupled to a bath comprising only a finite number of harmonic oscillators, which evolves in time as a closed Hamiltonian system, thus conserving information and energy.

Numerical solutions of the equations of motion reveal a rich dynamical scenario: For a single harmonic oscillator coupled to the double well, we observe Hamiltonian chaos emerging from integrable behaviour as predicted by the KAM theorem. Increasing the number of bath modes, the system comes closer and closer to an irreversible time evolution, replacing chaotic dynamics by relaxation into states that remain stable on increasingly long time scales. Being bistable and symmetric under spatial reflection, the long-time dynamics comprises two attractors, left and right minimum, which are approached with equal probability 0.5. Which one is reached, starting from an unbiased initial state of the central system on top of the barrier separating the minima, is reproducibly determined by the initial state of the environment.

With this behaviour, our model amplifies microscopic fluctuations to macroscopically observable randomness. Unlike Brownian motion, however, this stochastic process does not become manifest as a continuous quivering but as a stable discrete variable, a random binary number or a sequence of them if the trial is repeated. It keeps a lasting memory, encoding the initial state of the environment in a single bit. In this way, it reconciles the random outcomes of this toppling pencil experiment (analogous to tossing a coin) with two fundamental symmetries: It identifies the environment as the source of the entropy generated by the binary random sequence that violates the conservation of information in the macroscopic degree of freedom alone, and it explains how the parity symmetry of potential and initial state of the bistable system is broken by a microscopic bias in the initial state of the environment.

We hope that our work may serve as a template for further studies of discrete stochastic phenomena in systems that allow for a classical or semiclassical description, down to molecular physics. It remains an open question allows for any kind of conclusion concerning randomness in quantum systems. As a heuristic quantization of a double well coupled to a finite heat bath, we presently investigate the spin-boson model with a finite number of boson modes to provide some insight in this respect.

## Appendix A. Discretizing Frequencies according to a Given Spectral Density

We would like to construct a sequence of discrete frequencies $\omega_{n}$, n=1,…,N, that comply with a given density of states,

(A1) ρω(ω)dω:=∑nω≤ωn≤ω+dωδ(ω−ωn).

In this task, we have to cope with the difficulty that this functional dependence is defined with respect to the frequency ω, not to the actual independent variable, which in this context is a discrete index *n*, such as a quantum number counting energy eigenstates of a fictitious Hamiltonian. This requires to determine the functional dependence ω(x) on an independent variable *x*, continuous to begin with, so that, with an equidistant discretization of this variable,

(A2) $$x_{n}=x_{0}+n\Delta x,\;\Delta x=\frac{x_{N}-x_{0}}{N},\;n=1,\ldots ,N,$$

The frequencies $\omega_{n}=\omega \left(x_{n}\right)$ satisfy the required density of states $\rho_{\omega }\left(\omega \right)=\rho_{\omega }\left(\omega (x_{n})\right)$.

The function relating these two quantities, the frequency as a function of *x*

(A3) $$R^{+}∋x\mapsto \omega \left(x\right)\in R^{+},$$

results in a level density depending on *x*,

(A4) $$\rho_{\omega }\left(x\right)\;d\omega :=dn_{\omega }\left(x\right),$$

where $n_{\omega }\left(x\right)$ counts the number of discrete frequencies $\omega (x_{n})$ found between some lower bound $x_{0}$ and *x*. With the discretization (A2), assuming a sufficiently smooth function ω(x), it can be expressed in terms of the frequency step size or nearest-neighbour level separation,

(A5) $$\sigma_{n}:=\omega \left(x_{n}\right)-\omega \left(x_{n-1}\right)\approx {\left.\left(x_{n}-x_{n-1}\right)\frac{d\omega (x)}{dx}\right|}_{x=x_{n}}=\Delta x\omega^{\prime }\left(x_{n}\right),$$

as

(A6) $$\rho_{\omega }\left(x_{n}\right)\approx \frac{1}{\omega_{n}-\omega_{n-1}}=\frac{1}{\sigma_{n}}\approx \frac{1}{\Delta x\omega^{\prime }\left(x_{n}\right)}.$$

Taking the inverse of Equation (A6) and setting Δx=1, we obtain a general functional equation the function ω(x) has to satisfy,

(A7) $$\omega^{\prime }\left(x\right)=\frac{1}{\rho_{\omega }\left(\omega (x)\right)}.$$

Equation (A7) can also be understood as a direct consequence of the relation $\rho_{\omega }\left(\omega \right)d\omega =\rho_{x}\left(x\right)dx$, with $\rho_{x}\left(x\right)=const=1$, implied by Equation (A2) with Δx=1.

As an example, consider the algebraic density

(A8) $$\rho_{\omega }\left(\omega \right)=\Delta \omega^{-s-1}\omega^{s},\;\omega \ll \omega_{co},$$

(without cutoff, therefore not normalized), which includes the subohmic (s<1), the ohmic (s=1) and superohmic (s>1) regimes of solid-state physics [22,33]. Δω is a frequency scale introduced to make sure that $\rho_{\omega }\left(\omega \right)$ has the correct dimensionality $\omega^{-1}$. The differential equation,

(A9) $$\omega^{\prime }\left(x\right)=\Delta \omega^{s+1}{\left(\omega (x)\right)}^{-s}$$

(substituting Equation (A8) into Equation (A7)) is solved by

(A10) $$\omega_{n}={\left(s+1\right)}^{\frac{1}{s+1}}\Delta \omega \;n^{\frac{1}{s+1}},$$

so that the inverse spectral step size as a function of *n* becomes

(A11) $$\omega_{n}^{\prime }={\left(s+1\right)}^{\frac{-s}{s+1}}\Delta \omega \;n^{\frac{-s}{s+1}}.$$

Resolving Equation (A10) for n(ω), the level number as a function of the frequency,

(A12) $$n\left(\omega \right)=\left\lceil \frac{1}{s+1}{\left(\frac{\omega }{\Delta \omega }\right)}^{s+1}\right\rceil ,$$

substituted in Equation (A11), allows us to check Equation (A7) for the spectral density (A8),

(A13) $$\rho_{\omega }\left(\omega \right)=\frac{1}{\omega^{\prime }\left(n(\omega )\right)}={\left(s+1\right)}^{\frac{s}{s+1}}\frac{{\left(n(\omega )\right)}^{\frac{s}{s+1}}}{\Delta \omega }={\left(\Delta \omega \right)}^{-s-1}\omega^{s}.$$

In particular, for s=1 (Ohmic spectral density), see Figure A1a,b,

(A14) $$\omega_{n}=\Delta \omega \sqrt{2n}.$$

An exponential cutoff for large frequencies,

(A15) $$\rho_{\omega }\left(\omega \right)=\frac{1}{\omega_{co}}\exp\left(\frac{\omega }{\omega_{co}}\right),$$

Is obtained with the discrete spectrum, see Figure A1c,d,

(A16) $$\omega_{n}=-\omega_{co}\ln\left(N_{co}-n\right),\;\omega \gg \omega_{co},$$

Equations (A10) and (A16) have to be matched adapting the free parameters $\omega_{co}$ and $N_{co}$, with $N_{co}>N$, accordingly. A solution interpolating between Equations (A14) and (A16) is formally possible but not in closed analytical form.
